# Analyzing Llama 3-based Approach for Axiom Translation from Ontologies

**Published:** 2024-11

**Authors:** Xubing Hao, Licong Cui, Cui Tao, Kirk Roberts, Muhammad Amith

**Affiliations:** 1 McWilliams School of Biomedical Informatics, The University of Texas Health Science Center at Houston; 2 Department of Artificial Intelligence and Informatics, Mayo Clinic; 3 Department of Biostatistics and Data Science, University of Texas Medical Branch; 4 Department of Internal Medicine, University of Texas Medical Branch

## Abstract

Ontology development involves a top-down approach where ontology engineers and domain experts collaboratively define and evaluate ontological elements and axioms. Translating ontology axioms into natural language can significantly aid in ontology evaluation by making the content more understandable to subject matter experts who may lack a background in knowledge engineering. In this preliminary study, we investigate the potential of large language models (LLMs) in axiom translation from ontologies to facilitate ontology evaluation. We utilize Llama 3 to translate 1,192 ontology axioms across 19 distinct axiom types from five published ontologies. Results show that 163 (13.67%) of the Llama 3 translation of the axiom are accurately represented, 268 (22.48%) are not accurately represented, and 761 (63.84%) are partially accurate. Our manual evaluation of the Llama 3 translation indicates some competency in producing hierarchical natural language equivalents while revealing some limitations when translating complex axioms. Nonetheless, there are opportunities to improve the results with few-shot training or using LLMs to provide support in knowledge engineering for ontologies.

## 1. Introduction

In recent years, the immense amount of disparate and isolated databases, information systems, and knowledge sources have been developed across various domains. Ontology has emerged as a crucial resource within knowledge engineering, capable of addressing the bottleneck problems associated with managing and obtaining knowledge from these diverse sources [1]. Ontologies are a key component to the development and technologies of the Semantic Web [2, 3]. They play a vital role in information utilization through knowledge representation, and sharing and reuse [4]. The specific applications of ontologies include the creation of standardized conceptual vocabularies, providing services for queries, and developing reusable knowledge bases, all of which enhance interoperability across different systems [5].

Ontologies are modeled a defined domain space using interlinked triples (i.e., *subject* > *predicate* > *object*) [6]. Additionally, axioms are defined as assertions in a logical form, including rules that together comprise the overall theory that the ontology describes in its domain of application [7]. Axioms enable the definition of more complex relationships and constraints, adding depth and accuracy to the ontology’s representation. To ensure ontologies are machine-readable, special syntax is used to encode the interlinked triples. Commonly used languages for encoding the interlinked triples include the Resource Description Framework (RDF)/Terse RDF Triple Language (Turtle) [8], and Web Ontology Language (OWL) [9], a semantic enhanced extension of RDF.

Traditionally, ontology development involves a top-down approach where ontology engineers and domain experts collaboratively define ontological elements and axioms through iterative discussions and revisions [10]. This process can lead to omissions, redundancies, errors, and inconsistencies, making ontology evaluation an essential part of ontology development and maintenance [11]. Proper evaluation ensures that the ontology meets application requirements [11], enhances its availability and reusability [12, 13], and reduces maintenance costs for collaboratively created knowledge bases [12]. However, the terminology and methods surrounding ontology evaluation, especially in specialized fields, can be confusing and inaccessible to many researchers.

Translating ontology axioms into natural language can significantly aid in evaluation by making the content more understandable to subject matter experts who may lack a background in knowledge engineering, and the time and effort to navigate with ontology tools, such as Protégé [14, 15, 16]. Presenting ontologies in natural language helps subject matter experts better interpret the knowledge representation that can allow experts to effectively review and verify the information and provide valuable feedback to improve the quality and accuracy of the ontology.

Early approaches using Controlled Natural Language (CNL) such as Attempto Controlled English (ACE) [17] and Sydney OWL Syntax (SOS) [18] have been developed for primitive English representation of triples in an ontology model. However, CNL has the issues of ambiguity of text and is difficult to understand. To improve the clarity of the generated text, OWL ontology to natural language tools such as SWAT [19] and NaturalOWL [20] with linguistic fluency have been developed. Recently, researchers have made efforts on refining such approaches by removing repetitions and redundancies at the semantic level [21] and by making the verbalizer domain and schema independent [22]. Previously, we developed Hootation which is a software supporting precise natural language translation for 14 types of logical axioms in biomedical ontologies for the sole purpose for ontology evaluation by subject matter experts [16].

In recent years, Large Language Models (LLMs) have achieved advancement in Natural Language Processing (NLP) tasks, demonstrating their ability to capture complex language patterns across various domains of knowledge [23, 24]. Usage of LLMs has increased for human-centric tasks, and models like Generative Pre-trained Transformer (GPT) [25], and Large Language Model Meta Artificial Intelligence (LLaMA) [26] have attracted attention for different NLP tasks such as text classification, text generation, and question answering. To capitalize on the capabilities of LLMs, this study seeks to investigate their potential in axiom translation from ontologies to facilitate ontology evaluation.

## 2. Methods

### 2.1. Dataset

To ensure a diverse range of knowledge domains and ontology axiom types, this study utilizes five published ontologies encompassing 1,192 ontology axioms across 19 distinct axiom types that have been used in previous studies of our co-authors.

The People Ontology represents knowledge about various types of individuals, primarily based on familial information [16]. It serves as a teaching tool for introducing the development of OWL-based ontologies and the descriptive logic capabilities of OWL. The ontology comprises 13 classes and for the purpose of axiom translation in our approach, we retrieved 54 ontology axioms with 15 different axiom types.

The Social Determinants of Health (SDoH) Ontology represents knowledge of the social and economic characteristics of SDoH [27]. Concepts were gathered from 27 literature sources. The SDoH Ontology includes determinants at the macro, meso, and micro levels, covering topics such as health policy, welfare, work conditions, and gender. It comprises 383 classes and we retrieved 346 ontology axioms with 3 different axiom types for the purpose of our study.

The Ontology of Fast Food Facts (OFFF) normalizes and standardizes heterogeneous data sources of fast food information, facilitating the management of large volumes and rapidly changing nutritional data [28]. Constructed on metadata from 21 fast food establishment nutritional resources, OFFF includes 413 classes. For the purpose of our approach, we retrieved 457 ontology axioms with 6 different axiom types.

The Elements of Visuals Ontology (EVO) offers a comprehensive set of concepts and taxonomic structures designed to decompose visuals into basic elements [29]. As a fundamental-level ontology, EVO encompasses the essential aspects and elements involved in describing visualizations, such as shapes and colors. The ontology includes 943 classes and for our study purpose, we retrieved 182 ontology axioms with 14 different axiom types.

The Time Event Ontology (TEO) encompasses entities and definitions related to temporal information and their semantic relationships [16]. It formalizes temporal structures in structured data and textual narratives, providing core semantic components to represent temporal events and relations for enhanced reasoning. TEO includes 156 classes and we retrieved 153 ontology axioms across 9 different axiom types.

### 2.2. Model

Introduced by Meta in 2023, Llama is a collection of pre-trained and fine-tuned large language models that leverage an optimized transformer architecture, pre-trained through self-supervised learning on enormous text corpora [26]. In April 2024, Meta released Llama 3, featuring models with 8 billion and 70 billion parameters. With key improvements, Llama 3 has achieved state-of-the-art performance across a broad range of use cases [30]. In this study, we use the Llama-3–8B-Instruct which is Llama 3’s instruction fine-tuned variant with 8 billion parameters [31].

### 2.3. Prompt Design

In this study, we aim to assess Llama 3’s ability in translating ontology axioms into clear and accurate natural language phrases. We prompt Llama 3 with the question “Can you translate the ontology axiom to natural language?”. We provide the model with the ontology axiom type and the axiom itself, expecting it to generate a natural language translation of the ontology axiom. Table 1 presents a prompt using *FunctionalObjectProperty* axiom “⊤ ⊑ ≤ 1 hasGender” as an example.

### 2.4. Evaluation

We preform a qualitative review of the results where we examined the fidelity of the output with the actual axiom of the ontologies. If the translation accurately presents a sentence that is faithful to the axiom, we record Y for yes, N for inaccurate translation, and X for minimally accurate. For the latter, if the translation captures the essence of the axiom expression, yet, it may include terms or other information to the sentence that prevents it from being accurate.

Essentially our qualitative review consists of two parts - an *expression* assessment and *construction consistency* assessment. For every generated natural language axiom, we first assessed the fidelity of the natural language axiom with the notational axiom. If the assessment is inaccurate it is denoted as “N” (inaccurate). We then examine the construction and mapping of the natural language axiom to the notational axiom’s symbols and terminologies. In this stage, we want to ensure the consistency and that the labels are in accordance to the terms used by the axioms. For example, two translated axioms belonging to the same type can faithfully represent the axiom expressed, but how the terms are utilized (or if new terms are introduced) may differ. Therefore, a generated axiom that fails this stage may lead to an “X” (unknown) indicating an ambiguous production of the natural language translation. Otherwise, the produced natural language sentence is denoted as a “Y” (accurate).

## 3. Results and Discussion

Table 2 through 5 provide a breakdown of the review by entities (Table 3) and properties (object and data properties, Table 4 and 5, respectively). In total, we examined 1,192 logical axioms from the aforementioned ontologies (Table 2). We deduced that 163 (13.67%) of the Llama 3 translation of the axiom were accurately represented, 268 (22.48%) were not accurately represented, and 761 (63.84%) were partially accurate. Most of the axioms were of type SubClassOf (879), and similarly, most of the entity-related axioms (*SubClassOf*, *ClassAssertion*, *EquivalentClasses*, etc.) comprised of 77.27% (921) that were reviewed by us. Presumably, the translation of the data property axiom types accounted for 64.17% were accurate, and the object property axiom types had 69.97% that were clearly inaccurate. The entity type axioms had the least amount of inaccuracies (21.80%).

Our preliminary review of the results highlighted some potential strength and weakness of using an LLM for producing a natural language sentence from logical axioms of an ontology. In general, most of the Llama 3 translation were not directly accurate to the axioms that were encoded from the ontologies, with only 13.37% (163), however, majority of the translation were almost accurate despite some issues of the translation (63.84%, 761).

The translation failed for axiom types include *ObjectPropertyRange*, *DifferentIndividuals*, *SubObjectPropertyOf*, *TransitiveObjectProperty*, *InverseObjectProperty*, and *AsymmetricalObjectProperty*. For these types, none of the results were recorded as an accurate axiom translation (except for *ObjectPropertyRange* with only 2 accurate axiom translation). On a macro-level, the data property-related axioms (*DataPropertyRange*, *SubDataPropertyOf*, *DataPropertyAssertion*, and *FunctionalDataProperty*) tend to have better translations, despite the smaller sample. With translating object property-related axiom types, the model appears to perform poorly. The entity-related axiom types had a low number of incorrect translation in comparison to others. Potentially if we were to combine the partial (X) with the correct (Y), the accuracy results could be perceived as improved. One thing to note is that *SubClassOf* comprised the majority of axioms types pertaining to entities (879 to 42). The partial accuracies were due to added verbiage or rewording that closely captured the expression of the axiom. If these translation could be improved through training of the model, the fidelity to axiom translation could be improved. However, the foundational structure of many ontologies are hierarchies, which are simple “Every A is a B” expression. To translate this expression is relatively simple and can be accomplished with basic rule-based approach, which may beg the question of why go through the effort to train an LLM for a simple task? Ideally the model should be able to accomplish this task without the extra effort to train a model for a simple activity.

We also noted some other observations of the translations. One observation is the addition of content in “Secondary ⊑ Education_Level” where the translation was “A secondary level of education is a type of education level.” The precise translation is “every secondary is a education level.” The axiom had no context about what exactly is “Secondary” is, yet, the LLM-translation produced some added content and context. This type of translation, while not faithful to encoded axiom, gave some “explanation” of the axiom which could be of benefit to the knowledge engineer or to the subject matter expert reviewer. Yet the added content and context of the translation could produce unfaithful translation. For example, “∃ MSG.⊤ ⊑ Allergens” led to the generated translation of “There exists a message MSG such that MSG is about all allergens.” The model appears to mistaken MSG for “message”, instead of the food ingredient of MSG (Monosodium glutamate). We speculated that the underlying distributional semantic approach (a theory that posits models explain terms’ context by adjacent terms in corpora) trained on content where MSG is primarily found in context with “message”. However, one could assume that the knowledge engineer could have chosen a better label than MSG for the encoded fact in the ontology (i.e. blame the knowledge engineer).

Some cases we noted were some translations would miss key parts of the axiom while maintaining fidelity to the axiom expression. For example, “Sister ≡ (∃ hasSibling.Person) ⊓ (∃ hasGender.{female})” led to the LLM translation of “A person is a sister if and only if they have a sibling and are female.” The translation does capture the meaning and expression, but leaves out some information (e.g., “∃ hasSibling.Person”, “∃ hasGender.{female}”). Another example is “Stroke ⊑ (∀ hasCategoryWidth.WidthCategory) ⊓ (∀ hasLineCad.StrokeLineCap) ⊑ (∀ hasStrokeColor.Color)” which lead to “A stroke is a type of stroke that has a width category, a line cap, and a color” that is simpler yet missing some information from the axiom, similar to what was described in the other example.

While an early preliminary nature of this work, we do acknowledge some limitations of this work. One clear limitation is the unbalanced dataset used. For example, the ontologies used varied in their size as well as their utilization of different OWL2 features. Also the diversity of the axiom types skewed more for *SubClassOf*. This hierarchical axiom (“type of”) is more or less the foundational structure of all ontologies, and would undoubtedly be largely represented in this dataset. Ideally, however, to attain a more balanced evaluation of the performance of the LLM, it would be beneficial to generate a large sample of the other axiom types. From an LLM perspective, it is possible that the results of the translation may have been influenced by the prompt. In the future, we could refine and test the “wording” of the prompt and also conduct few shot learning or fine-tuning to determine an optimal result. Finally, our results are limited to Llama 3 model, and it may be possible the translation may differ with a different LLM. In the future, we plan to experiment with various LLMs and compare their performance. Additionally, we aim to evaluate their performance against baseline methods to assess the accuracy and efficiency of LLMs in axiom translation.

Despite some of the challenges discussed, there are some revealing opportunities to use large language models for axiom translation. One interesting use-case is the possibility of using a large language model as an “assistant” for ontologists to provide better labels and annotations for the entities and properties. There were cases where the translation, provided additional verbiage to the resulting sentences. In that circumstance, a knowledge engineer could re-evaluate the description of the entities to enhance the label construction for the entities. Another opportunity is for subject matter experts reviewing the ontology. When using Hootation, we provide the spreadsheet of the axiom translation to subject matter experts who are domain experts in their respective fields (physicians, public health experts, etc.). From those past experiences, the precise “existential” language of the translation (while faithful to the axiom) may baffle and confuse the reviewers. In certain results, we realized that large language models-based translation could be used as a contextual explanation to complement the actual translation. Overall there are obvious functions where LLMs could be used to assist in the knowledge engineering of ontologies.

Earlier we discussed the need for a varied data set where other axiom types have a significant sample size. A future possible direction is to further extend this work to review a reasonable sample of axiom types. This effort would help in providing a more comprehensive analysis and possible outcomes in how to better leverage an LLM, specifically Llama 3 for axiom translation of ontologies. With recent attention for LLMs to perform natural language processing on data, we are interested in integrating open-sourced LLMs, like Llama 3, in our development work for Hootation. With some of the preliminary findings, we are currently working on integrating LLM models to possibly enhance the knowledge engineering and evaluation experience like enhancing the label choices or providing some other functional roles like context provision. Lastly, our early preliminary work utilized Llama 3, and therefore we will investigate other open-source LLMs for future analysis and evaluation to extend this work.

## 4. Conclusion

In this study, we examined some early attempt to use an LLM (specifically Llama 3) to translate logical axioms from sample published ontologies using their past evaluation data. Results of the Llama 3 translation indicate some competency to produce hierarchical natural language equivalents. However, there are some limitations when translating complex axioms. This may be due to limitations of the underlying distributional semantics from learning the context of terms in the training data. Nonetheless, there are opportunities to improve the results with few-shot training or using LLMs to provide support in knowledge engineering for ontologies.

## Figures and Tables

**Table 1:** Prompt Design for Different Tasks.

Task	Prompt
Ontology axiom translation	<user> ### Can you translate the ontology axiom to natural language? ### Axiom Type: FunctionalObjectProperty ### Axiom: ⊤ ⊑ ≤ 1 hasGender ### Natural Language Translation (Only state your translation): </user>

**Table 2:** Overview of the Review of the Logical Axioms.

Y	N	X	Total
163 (13.67%)	268 (22.48%)	761 (63.84%)	1,192

**Table 3:** Review of the Entity-related Axioms.

Y	N	X	Total
81 (9.22%)	71 (8.01%)	624 (82.70%)	879 (SubClassOf)
15 (88.24%)	2 (11.75%)	0	17 (DisjointClasses)
2 (22.22%)	0 (0%)	7 (77.78%)	9 (EquivalentClasses)
5 (33.33%)	10 (66.67%)	0	15 (ClassAssertion)
0	1 (100%)	0	1 (DifferentIndividuals)
33.34%	21.80%	44.87%	921 (77.27%)

**Table 4:** Review of the Object Property Type Axioms

Y	N	X	Total
2 (2.33%)	75 (87.21%)	9 (10.47%)	86 (ObjectPropertyRange)
11 (15.71%)	51 (72.86%)	8 (11.43%)	70 (ObjectPropertyDomain)
8 (88.89%)	0	1 (11.11%)	9 (ObjectPropertyAssertion)
4 (36.36%)	4 (36.36%)	3 (27.27%)	11 (SymmetricalObjectProperty)
2 (66.67%)	1 (33.33%)	0	3 (FunctionalObjectProperty)
0	10 (100%)	0	10 (SubObjectPropertyOf)
0	5 (100%)	0	5 (TransitiveObjectProperty)
0	1 (100%)	0	1 (InverseObjectProperties)
0	1 (100%)	0	1 (AsymmetricObjectProperty)
23.33%	69.97%	6.70%	196 (16.44%)

**Table 5:** Review of the Data Property Type Axioms.

Y	N	X	Total
18 (56.25%)	9 (28.13%)	5 (15.63%)	32 (DataPropertyDomain)
11 (37.93%)	17 (61.54%)	1 (3.45%)	29 (DataPropertyRange)
1 (100%)	0	0	1 (SubDataPropertyOf)
1 (100%)	0	0	1 (DataPropertyAssertion)
4 (26.67%)	11 (73.33%)	0	15 (FunctionalDataProperty)
64.17%	32.02%	3.81%	78 (6.54%)
